# Orthodontic rubber band combined with through-the-scope twin clip-assisted endoscopic intermuscular dissection for large laterally spreading tumors in the distal rectum

**DOI:** 10.1055/a-2686-2701

**Published:** 2025-09-05

**Authors:** Kunming Lv, Xiangdong Wang, Yifan Xu, Yaoqian Yuan, Enqiang Linghu, Qianqian Chen

**Affiliations:** 1 670131 Department of Gastroenterology, The Second Medical Center of Chinese PLA General Hospital, Beijing, China; 2 651943 Department of Gastroenterology, The First Medical Center of Chinese PLA General Hospital, Beijing, China; 3 694508 Department of Gastroenterology, General Hospital of Central Theater Command, Wuhan, China

Endoscopic intermuscular dissection (EID) is a critical therapeutic approach for early gastrointestinal cancers and precancerous lesions 1 . However, in cases involving large laterally spreading tumors (LSTs) in the distal rectum, two persistent challenges compromise surgical outcomes: inadequate intraoperative visualization due to limited surgical field exposure and optimal closure of extensive post-resection defects 2 3 . While conventional dental floss-assisted traction offers procedural simplicity, it often fails to provide sufficient traction force during EID for large distal rectal LSTs, resulting in incomplete submucosal layer exposure 4 . Meanwhile, the closure of large mucosal defects postendoscopic resection poses a significant challenge. Traditional methods, including the titanium clip and the over-the-scope clip, have limitations in terms of complexity and applicability to large defects 5 . To address these limitations, we innovatively integrated orthodontic rubber band-assisted traction with the through-the-scope twin clip, performing a stepwise non-full-thickness super minimally invasive resection (SMIS). This combined technique not only enhanced submucosal muscular layer visualization but also enabled rapid and secure closure of large postresection defects. Characterized by operational simplicity and cost-effectiveness, this approach represents a novel solution for managing extensive LSTs in the distal rectum.

A 41-year-old man was admitted to our hospital with a long diameter of approximately 5-cm granular laterally spreading tumors (LSTs-G) in the distal rectum. We used a stepwise non-full-thickness SMIS for treatment ( Video 1 ). The surgical steps were as follows: first, an electrocautery was used to circumferentially mark the edge of the lesion ( Fig. 1 **a** ), and submucosal injection was performed to fully lift the lesion. Then, the electrocautery was used to incise the mucosal layer around the lesion ( Fig. 1 **b** ). After peeling off part of the submucosa, the orthodontic rubber band was used to fix the peeled-off anal-side mucosal layer of the lesion and pull it to the opposite mucosal layer on the oral side ( Fig. 1 **c, d** ), thus achieving a clear separation between the muscularis mucosae and the submucosa ( Fig. 1 **e** ). Subsequently, a triangular knife was applied to peel along the superficial layer of the muscularis propria, and the lesion was finally successfully and completely resected ( Fig. 1 **f** ). The size of the defect was approximately 7.0 cm, and the longitudinal muscular layer was visible. After hemostasis, through-the-scope twin clips combined with titanium clips were used to close the defect ( Fig. 1 **g** ). The size of the specimen measured outside the body was approximately 5.0 cm × 3.0 cm ( Fig. 1 **h** ). Pathological examination of the specimen identified a rectal tubular-villous adenoma with focal high-grade intraepithelial neoplasia (HGIN) and a small focus of intramucosal carcinoma. Both the lateral and deep margins were negative ( Fig. 1 **i** ). No adverse events occurred, with 3 months postoperative colonoscopy confirming a well-healed resection site ( Fig. 1 **j** ).

Application of orthodontic rubber band combined with through-the-scope twin clip in endoscopic intermuscular dissection for managing extensive laterally spreading tumors in the distal rectum.Video 1

**Fig. 1:**
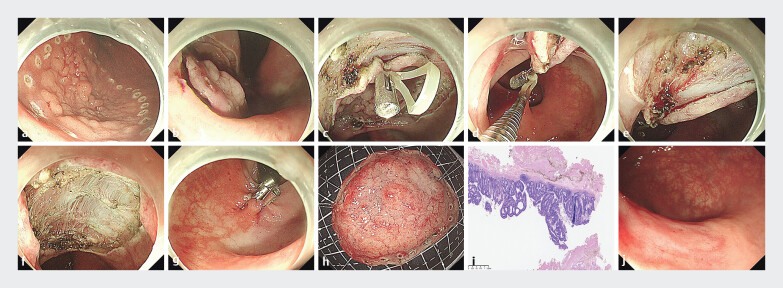
The operation steps of super minimally invasive surgery for the LSTs. **a** Circumferential marking of the lesion. **b** Electrocautery incised the mucosa circumferentially around the lesion. **c** The orthodontic rubber band was fixed to the anal-side mucosa of the lesion using titanium clips. **d** The anal-side mucosa of the lesion was retracted to the mucosa opposite the oral-side margin of the lesion. **e** Achieving a clear separation between the Muscularis mucosae and the submucosa. **f** The lesion was finally successfully and completely resected. **g** The defect was completely closed using a combination of through-the-scope twin clips and clips. **h** The specimen of the LSTs. **i** Rectal tubulovillous adenoma with focal HGIN and intramucosal carcinoma, margins negative. **j** Healed mucosal scar at 3 months post-operative colonoscopy.

This case fully demonstrates the advantages of the technique of orthodontic rubber band traction assistance combined with through-the-scope twin clip in EID for the treatment of large laterally spreading tumors in the low rectum. With its elasticity and simplicity, the orthodontic rubber band provides continuous and stable traction force for the operation, contributing to the smooth progress of the surgery. At the same time, the application of the through-the-scope twin clip achieves the safe closure of a large defect. With its advantages such as convenient operation and remarkable therapeutic effects, this technique is expected to become the preferred option for endoscopic treatment of large lesions in the distal rectum, and it has important clinical promotion value.

Endoscopy_UCTN_Code_TTT_1AQ_2AD_3AD

## Notes

Endoscopy E-Videos https://eref.thieme.de/e-videos
*E-Videos* is an open access online section of the journal *Endoscopy* , reporting on interesting cases and new techniques in gastroenterological endoscopy. All papers include a high-quality video and are published with a Creative Commons CC-BY license. Endoscopy E-Videos qualify for HINARI discounts and waivers and eligibility is automatically checked during the submission process. We grant 100% waivers to articles whose corresponding authors are based in Group A countries and 50% waivers to those who are based in Group B countries as classified by Research4Life (see: https://www.research4life.org/access/eligibility/ ). This section has its own submission website at https://mc.manuscriptcentral.com/e-videos .
